# Metal–Organic Frameworks in Modern Physics: Highlights and Perspectives

**DOI:** 10.1002/advs.201900506

**Published:** 2019-07-18

**Authors:** Yuri A. Mezenov, Andrei A. Krasilin, Vladimir P. Dzyuba, Alexandre Nominé, Valentin A. Milichko

**Affiliations:** ^1^ Faculty of Physics and Engineering ITMO University St. Petersburg 197101 Russia; ^2^ Ioffe Institute St. Petersburg 194021 Russia; ^3^ Institute of Automation and Control Processes FEB RAS Vladivostok 690041 Russia; ^4^ Université de Lorraine Institut Jean Lamour UMR CNRS 7198 Nancy F‐54011 France

**Keywords:** artificial structures, energy transfer, memory, metal–organic frameworks, particle physics

## Abstract

Owing to the synergistic combination of a hybrid organic–inorganic nature and a chemically active porous structure, metal–organic frameworks have emerged as a new class of crystalline materials. The current trend in the chemical industry is to utilize such crystals as flexible hosting elements for applications as diverse as gas and energy storage, filtration, catalysis, and sensing. From the physical point of view, metal–organic frameworks are considered molecular crystals with hierarchical structures providing the structure‐related physical properties crucial for future applications of energy transfer, data processing and storage, high‐energy physics, and light manipulation. Here, the perspectives of metal–organic frameworks as a new family of functional materials in modern physics are discussed: from porous metals and superconductors, topological insulators, and classical and quantum memory elements, to optical superstructures, materials for particle physics, and even molecular scale mechanical metamaterials. Based on complementary properties of crystallinity, softness, organic–inorganic nature, and complex hierarchy, a description of how such artificial materials have extended their impact on applied physics to become the mainstream in material science is offered.

## 1 Introduction

**Figure 1:**
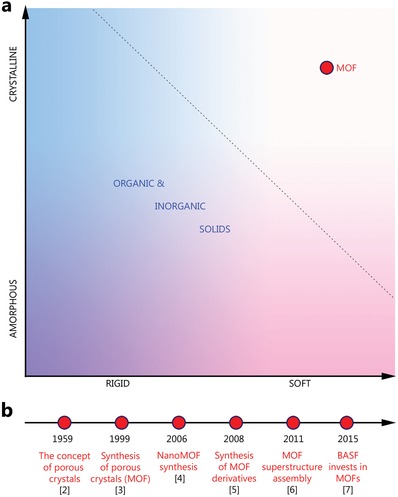
a) Metal–organic framework as a unique material with combination of crystallinity and softness. b) Developing of technologies for MOF synthesis and modification from an idea to real‐world application.

Following their conception more than 60 years ago, porous crystals have recently been synthesized in the form of metal–organic frameworks (MOFs), one of the key materials in modern chemistry and the chemical industry (**Figure** **1**).^1^ The development of this field of science is owed to the development of reticular chemistry, which enabled the acquisition of different complex structures that maintain operation in real conditions, for real‐world application.^2^, ^3^, ^4^, ^5^, ^6^, ^7^ At the same time, MOFs themselves have stimulated the development of a new field of science, in the form of computational chemistry, in response to problems encountered in finding optimal structures,^8^ which allows researchers to form a logical circle from the statement of the required property, through the design of the structure, and its synthesis to application.

The simple, yet, intriguing idea of an artificial porous crystalline material with dynamic response to external stimuli^9^ has also found partial interest in other areas such as nonlinear optics,^10^ biology, and medicine.^11^, ^12^ However, most existing MOFs, and design of MOFs, has been chemically oriented until now. Even so, focusing on the very concept of a porous crystal, one can ask the following question: Can MOFs be the key materials in other disciplines of science?

The answer lies in the combination of complementary properties of crystallinity, softness, organic–inorganic nature, and complex hierarchy. Today, the potential implementation of MOFs in applied science goes far beyond the structure‐related properties that are usual for most materials. Moreover, this combination opens up prospects for applied and fundamental science that have not yet been conceived.

**Figure 2:**
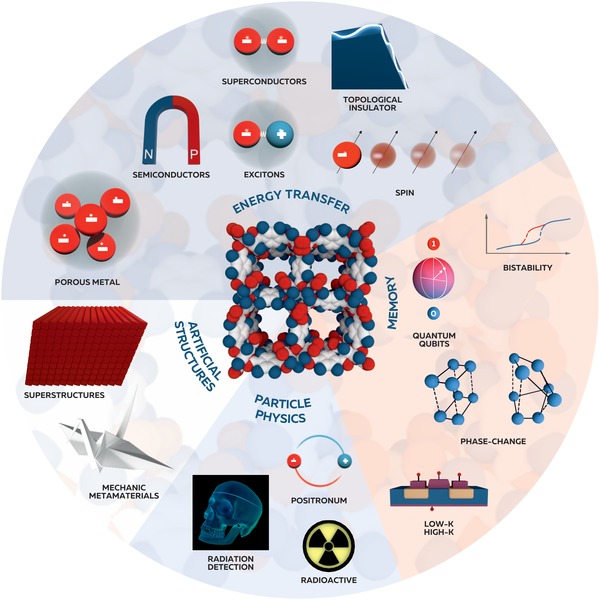
Illustration of application of MOFs in modern physics. Superstructures reproduced with permission.^[135]^ Copyright 2019, Springer Nature.

In this article we consider MOFs as a new family of functional materials in modern physics, reviewing topics ranging from porous metals and superconductors, topological insulators, classical and quantum memory elements, to optical superstructures, materials for particle physics, and even molecular scale mechanical metamaterials (**Figure** **2**). Within each section we also describe the current state of research and the perspectives for utilizing MOFs as i) an active substrate for energy and information transfer by electrons, excitons, photons, and spins, ii) active and passive materials for classical and quantum memory devices and triggers, iii) active materials for emission and detection of high‐energy particles, and iv) as hierarchical optical superstructures and mechanical metamaterials.

## 2 Transfer Effects in MOFs

**Figure 3:**
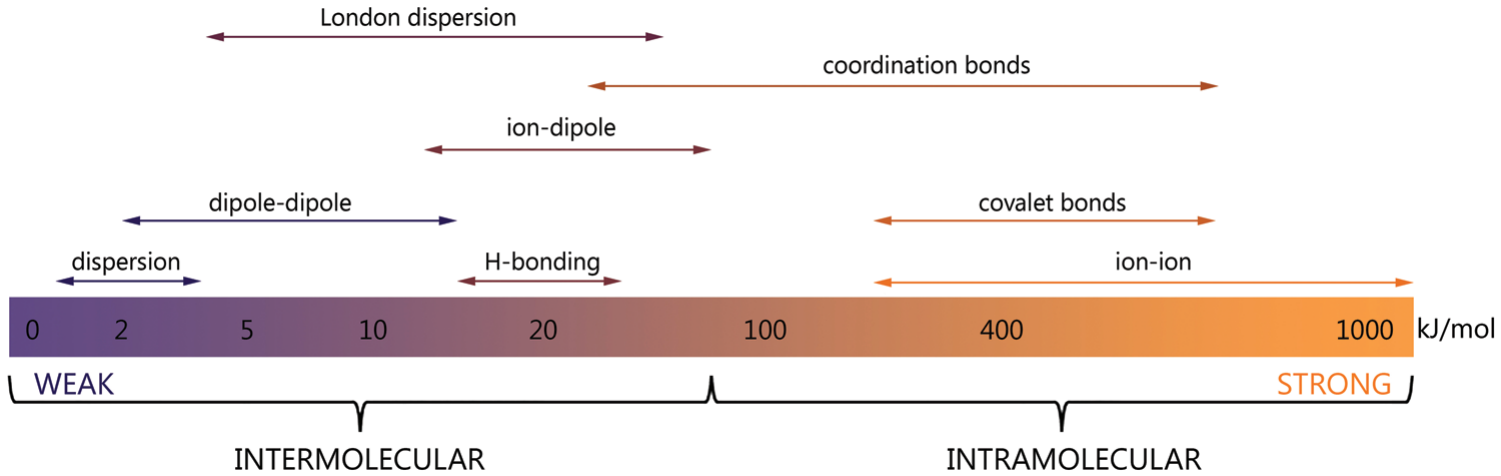
Energy scale of weak (dispersion, van der Waals, hydrogen, etc.) and strong (covalent, coordination, etc.) interactions between MOF building blocks.

Metal–organic frameworks are a novel class of molecular crystal with nonconventional inner structure based on the weak (dispersion, van der Waals, hydrogen, Casimir forces, etc.) and strong (covalent, coordination) interactions between building blocks (organic ligands, metal nodes, and solvent molecules) and structural units such as layers and chains (**Figure** **3**). The energy spectrum of charge carriers and complicated 1D, 2D, and 3D structures allow MOFs to have more diverse electronic states than ordinary crystals. These states can provide efficient energy transfer within a single crystal,^13^ a key point for the purposes of solar energy, microelectronics, transistors, batteries, supercapacitors, and other applications.

In this section, we discuss the highlights and perspectives for implementation of MOFs as active elements for energy transfer in microelectronic and optical devices due to their metallic, semiconducting, superconducting, topological, excitonic, and magnetic properties. We intentionally omit the effect of proton transfer, since it has been extensively reviewed in recent years.^14^

### 2.1 Semiconductors

Semiconductors are solids exhibiting a bandgap within the spectrum of electronic states (≈0.1–3 eV), and a relatively high electric conductivity that is due to a high concentration of free charge carriers with high mobility compared to dielectrics. The small bandgap, along with the concentration of free charge carriers, makes the conductivity of semiconductors strongly dependent on temperature, impurities, radiation effects, and electromagnetic fields in the visible and infrared frequency ranges. Semiconductor materials play a key role in modern microelectronics and optics, and metal–organic frameworks have recently emerged as a new family of semiconducting crystals,^15^ demonstrating the most complex structure of five or more chemical elements.

To make MOFs applicable as semiconductors, one must design the bandgap structure (direct or indirect), lower the bandgap, and/or increase the charge mobility. Metal nodes strongly affect the electric conductivity of semiconducting MOFs.^16^ For instance, structures based on iron nodes in a high energy state with mixed valency (Fe^2+^ and Fe^2+/3+^), exhibit better conductance compared to structures based on others metal nodes (Mn, Co, Mn, Ni, Cu, Zn).^17^ This phenomenon is explained by the large radius and the low effective core charge of the Fe^2+^ ion, while valence electrons have a weak bond with the core. While these valence electrons have a high energy that leads to the emergence of a low bandgap (2.0–2.2 eV), there is still not a clear understanding of how this process operates. Metals with higher d‐electron numbers, such as Fe, Co, Ni, Zr, and Hf, are predicted to significantly increase the energy of the Fermi level.^18^ As such, the metallic d band has an influence on the transport properties of MOFs.^19^

Another intriguing property is the fact that dimensionality can also affect the electric conductivity; some 3D and 2D MOFs exhibit different values of electronic conductivity.^20^ For instance, 2D and 3D MOFs Ni_3_(2,3,6,7,10,11‐hexaiminotriphenylene)_2_ have conductivities of 2 and 40 S cm^−1^, respectively. Currently, the achieved value of electric conductivity of some 2D^21^ and 3D^22^ MOFs is comparable to that of the classical inorganic solids (>1000 S cm^−1^ and >1 S cm^−1^, respectively), making MOF expected to become one of the important materials in semiconducting technology. Moreover, MOFs remain conductive under a range of excitation wavelengths, from ultraviolet to short‐wavelength infrared light (300–2500 nm).^23^, ^24^ MOFs with stable, accessible, and dense active sites, developed for high‐power energy storage devices through conjugative coordination between a redox‐active linker (2D MOF Co‐hexaaminobenzene),^25^ can also exhibit high electrical conductivity.

Due to their high porosity, low density, tuneable structures, and water resistivity for some compounds,^26^ as well as the development of thin film technology,^27^, ^28^ metal–organic frameworks represent promising semiconductors^29^ for application as charge transfer materials for advanced semiconducting devices, microelectronics, elements for energy storage, and batteries.

### 2.2 Metals

Metallic conductivity is an unexpected feature of metal–organic frameworks, discovered very recently in 2D crystals.^30^ With semiconducting MOFs, the metallic effect is determined by strong overlapping of the electronic wave functions between metal nodes and ligands, making π‐conjugation essential for a metallic nature. The high degree of such π‐conjugation, turned on by external stimuli such as voltage, light, and temperature, provides MOFs with metallic conductivity. However, the origins of this effect are obscure, as it is observed only with 2D MOFs;^30^, ^31^, ^32^, ^33^, ^34^, ^35^ MOFs usually demonstrate semiconducting properties between 170 and 300 K, while they are metals below 130 K, as in a 2D MOF such as cobalt 2,3,6,7,10,11‐triphenylenehexathiolate.^30^ Moreover, MOFs can demonstrate bimodal charge transport: metallic in the plane of the MOF's van der Waals (vdW) layers, and semiconducting normal to these layers.^33^ This issue raises the question of how suitable MOF structures demonstrating metallic conductivity can be predicted.^34^ One of the latest investigations consisted of combining machine learning methods and ab initio calculations to predict the metallic nature of MOFs. Based on density‐functionaltheory (DFT) band theory, it is predicted that six MOF structures (Mn_8_Re_24_C_24_S_32_N_24_, Mn_8_Re_24_C_24_Se_32_N_24_, Mn_8_Re_24_C_24_Te_32_N_24_, Co_4_Hg_4_C_16_S_16_N_16_, Cd_2_C_8_, Mn_4_Re_12_Te_16_C_12_N_12_) should have metallic behavior, while a monolayer of Cu‐benzenehexathial is expected to exhibit higher conductivity.^35^ This behavior is explained by the crossing of several bands with the Fermi level, indicating the intrinsic metallic nature of the MOF.

Metallic porous crystals can be considered as the first artificial porous metals made by reticular chemistry.^36^ Such unusual crystals, demonstrating the efficient transfer of electrons, can be applied to future solar cells, fuel cells, batteries, and microelectronic devices. A further goal for developing this new type of metal is the synthesis and study of MOFs that will work at room temperature with giant electric conductivity compared to common metals, as at present, the achieved conductivity is one order of magnitude smaller.

### 2.3 Superconductors

Superconductive materials exhibit the largest degree of conductivity of all the materials. The phenomenon is of a purely quantum nature, and arises due to the binding of free charge carriers into pairs with the whole spin, with further transition to the Bose condensate state. This transition is possible at specific temperatures and external magnetic fields below a critical value, and is accompanied by a strong decrease in electrical resistance, even going to zero in the case of direct currents. Although only a small number of organic (polysulfur nitride)^37^ and inorganic superconductive materials^38^ have been discovered, 2D and 3D MOFs could potentially extend this family, as shown by Liu and coworkers.^39^ The calculation revealed that 2D in‐plane coordinations, such as Cu—S, Cu—O, and Fe—Se bonding networks, play the crucial role in triggering superconductivity in systems like Cu‐benzenehexathial. This particular quality explains the high conductivity in Cu‐benzenehexathial.

This prediction can stimulate the field of hybrid superconductors because such MOF materials can be synthesized on liquids and surfaces with the desired morphology, to be applied for high‐energy physics, charge transport, and superconducting microwave devices of the future.

### 2.4 Excitons

**Figure 4:**
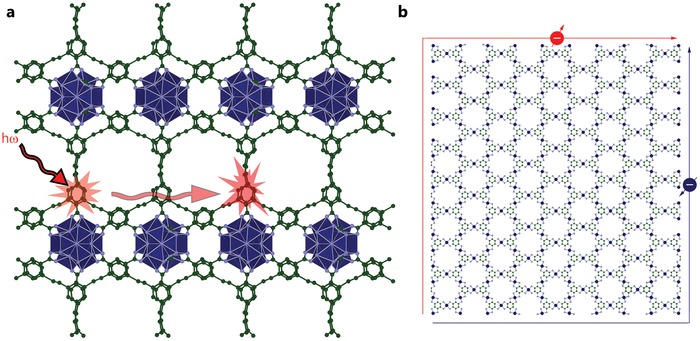
a) Illustration of generation of exciton by light and its transfer within the 2D MOF. The structure was extracted from crystallographic information file (CIF).^44^ b) Illustration of opposite transfer of electrons with opposite spins within the surface of 2D MOF demonstrating topological states. The structure represents the one published in ref. 67.

An exciton is a Coulomb bound between an electron and a hole. For excitons, the quantum states of electrons and holes are correlated, and they are thus considered as a single particle that has a hydrogen‐like energy spectrum lying within the bandgap. The minimum light energy required for the generation of an exciton is equal to the difference between the width of the bandgap and the exciton binding energy. For molecular crystals and low‐dimensional structures, the binding energy of an exciton can be much higher than the thermal energy (0.026 eV). Such a high binding energy (>0.1 eV), allowing the exciton to exist at room temperature, is a key aspect for applications such as excitonic lasers,^40^ valeytronics,^41^ and organic solar cells.^15^, ^42^ Moreover, the exciton is such a specific particle that its migration within a structure (**Figure** **4**a) can be driven by external stimuli or structural anisotropy.^43^ Exciton complexes, such as biexcitons, and indirect excitons, can also be manipulated by optical, electric, and magnetic fields and tuneable structures.

The use of MOFs as molecular crystals for exciton generation appears to be unprecedented.^44^, ^45^, ^46^, ^47^, ^48^, ^49^, ^50^, ^51^, ^52^, ^53^, ^54^ Rational design of 2D and 3D MOFs can provide excellent exciton transport,^45^ a phenomenon that is most efficient for MOFs which contain chromophore‐based ligands separated by 1–2 nm distances. Such ligands serve as antenna‐like parts that collect light energy. In this way, long distance exciton migration within the MOF occurs in a weak electronic coupling regime. Suitable MOF designs^54^ ensure that the exciton energy can be transferred from several tens to several hundred nanometers.^45^, ^47^ Similar to their metallic properties, the remarkably efficient exciton migration within MOFs can be attributed to enhanced π‐conjugation obtained through the addition of some moieties (e.g., acetylene), which leads to greater absorption of light energy and much faster exciton energy transfer.^46^

It has been shown that energy transfer by excitons can be described by a Dexter triplet‐to‐triplet (exchange) mechanism with multistep incoherent exciton hopping, or a Förster mechanism involving one‐step jumping over longer distances.^47^ Moreover, the rate and efficiency of the energy migration for MOFs such as Tb_0.95_Eu_0.05_HL (HL = 5‐hydroxy‐1,2,4‐benzenetricarboxylic acid) can be significantly improved by mixing different ions (multivalence).^55^ Intriguingly, the structure of MOFs also affects the exciton migration path, as it was discovered that mixed Ru/Os structures constituted 1D or 3D hopping networks.^45^

The existence of excitons at room temperature, and their ability to transfer energy within the MOFs, is important for solar energy and excitonic devices.^42^ An ability to generate excitons by light, and electrical control of these excitons, also supports MOF application in optoelectronics in the near future,^56^ and we believe that MOFs can compete with materials such as inorganic 2D vdW crystals and heterostructures,^57^ organic polymers, and crystals for micro‐ and nano‐electronic devices that operate based on the Bose–Einstein condensation effect.^58^, ^59^ However, there are problems to be solved prior to this application, as an increase in the monocrystallinity^60^ and size of single MOF crystals, increases the binding energy, rates of exciton hopping and energy transfer.

### 2.5 Spin Transfer

The spin transport effect underlies the field of spintronics, which concerns the application of the spin of an electron to the delivery of information.^61^ This opens up new possibilities for new‐generation devices that combine conventional microelectronics with spin‐dependent effects. Metal–organic frameworks demonstrate the spin transfer effect because they possess different magnetic properties at low temperature. As with their semiconducting, metallic, and excitonic properties, the MOF's structure determines this spin transfer effect; spin transport is observed mostly in 2D MOFs (e.g., M_3_C_12_S_12_ and M_3_C_12_O_12_, where M = Zn, Cd, Hg, Be, or Mg).^62^ Such MOFs exhibit double Dirac cones (quantum mechanical features occurring in electronic band structures and describing unusual electron transport properties) with different Fermi levels. The crossing of two cones leads to two independent channels akin to spin channels in spintronics. For M_3_C_12_O_12_ with conjugated metal‐tricatecholate polymers M_3_(HHTP)_2_ (where HHTP is 2,3,6,7,10,11‐hexahydroxytriphenylene), the spin‐polarized slow Dirac cone center is pinned precisely at the Fermi level, making the systems conducting in only one spin/cone channel. It should be noted that spin liquid states (the liquid behavior of spins at low temperatures) have also been recently observed with MOFs.^63^, ^64^ These special states being extremely complex to observe experimentally, MOFs could be a platform adapted for spintronics. The ability for spin transfer can make MOFs with specific magnetic properties promising materials for spintronic devices such as spin FETs (field‐effect transistors), spin LEDs (light‐emitting diode), spin RTDs (resonant tunneling devices), terahertz optical switches, and quantum computation and communication.

### 2.6 Topological Insulators

Topological insulators are a new kind of quantum material with specific electronic states (the bulk is an insulator while the edges are a semiconductor or metal) induced by the topology of the band structure.^65^ Although the existence of such states has already been confirmed for limited numbers of inorganic compounds and artificial metamaterials,^66^ no experimental data exists for organic, and especially, metal–organic materials. In this case, the calculation approach^31^ also helped to reveal that MOF structures satisfy the requisite topological concept (Figure 4b); since 2013, there have been several theoretical works based on 2D structures confirming the existence of topological states allowing an opposite transfer of electrons with opposite spins.^67^, ^68^, ^69^, ^70^, ^71^ These states should originate from electrons filling the hybridized bands of metal ions and molecular orbitals of the ligands.^68^ The unconventional electronic states of MOFs make such materials promising for nontrivial energy/information transfer by electrons (spins) or photons (polarization) in a new generation of electronic devices based on spintronic or quantum computing principles.

## 3 Memory Effects in MOFs

The soft,^9^ porous, and organic–inorganic nature of MOFs enable the utilization of these crystalline materials for next generation memory and trigger devices. Due to their softness, MOFs demonstrate dynamic reversible and nonreversible structural changes in response to external stimuli such as pressure, electric and magnetic fields,^72^, ^73^ and light,^74^ and can be implemented as active binary systems for recording information. The organic nature of MOFs provides specific dielectric properties (low‐*k* or high‐*k*) which support application as passive elements in transistors and microcircuits. Furthermore, their complex organic nature (bonded ligands and solvents) paves the way for specific structural transitions that are important for ultrafast optical data processing and storage devices of the near future. Conversely, the inorganic nature of MOFs provides unique states such as quantum qubits that can also be utilized for recording information with unprecedented density. Here we discuss the perspectives of classical and quantum memory and trigger devices based on MOFs.

### 3.1 Low‐*k* and High‐*k* Dielectrics

Dielectric permittivity describes the response of matter to an external electric field. This response is also characterized by the relative permittivity, which describes the influence of an external electric field on electromagnetic processes inside a material. For weak excitation, this influence can be considered linearly dependent on the strength of the external electric field. In case of strong excitation at optical frequencies, the dielectric permittivity becomes dependent on the speed of electric field variation. High‐*k* and low‐*k* materials refer to materials with high and low dielectric constants (i.e., relative permittivity), respectively. The latter are commonly used in microelectronic circuits and integral cells as triggers.^75^ The main issues that confront such electronic elements are i) miniaturization, ii) increasing the speed of information processing, and iii) improving the flow of an electrical signal. However, the most important problem is time delay when an electrical signal flows from one element to another. Microelectronic devices are more efficient if the delay time is shorter, and the use of low‐*k* materials helps to improve this parameter. In contrast, dielectrics used in transistors should demonstrate a high polarization effect (i.e., high‐*k* feature) for efficient data processing.^76^

In case of a low dielectric constant the reducing of the number of dipoles or their strengths should be achieved. Therefore, it is logical to use those connections in materials that are nonpolar, such as C–C or C–H.^75^ One such example is the family of zeolitic imidazolate frameworks (ZIF‐4, ZIF‐7, ZIF‐8, ZIF‐71, and ZIF‐90). THz‐frequency dielectric constants are dependent on structure, and have a strong correlation with the porosity and density of the MOF. The dielectric constant in the near infrared region (>4000 cm^−1^, i.e., >120 THz) is ≈1.2 for most structures, which corresponds to low‐*k* materials (≈1.2–2.5). The only exception is ZIF‐90, whose higher dielectric constant (≈1.5) is likely due to microstructural defects.^77^ Experiments with films based on different thicknesses of ZIF‐8 (400–1000 nm) also yielded low dielectric constants of 2.33 ± 0.05 at 10^5^ Hz.^78^

Varying the composition of the MOF allows the high‐*k* property to be achieved. This is mainly due to the small gap between the valence and conduction bands, where there exists a large admixture of excited electronic states relative to the ground state when an electric field is applied. This leads to high polarizability and, accordingly, large dielectric constants. Also, the use of transition metal ions (V, Cr, Zr, Mo) increases the ionic and electronic polarizability due to the presence of d‐electrons with anisotropic electronic‐spatial distributions.^72^ The film and bulk versions of the (Zn(TBTC)[H_2_N(CH_3_)_2_]}·2DMF·EtOH) MOF recently exhibited high dielectric constants (19.5 and 5.9, respectively) at a frequency of 1 MHz. This is likely due to the interpenetrating nature of MOF film deposited on conductive substrate, while with the bulk, it can be explained by the polarization of the packed microstructure of the MOF due to the solvent molecules. MOF films have a high mechanical flexibility and can withstand high mechanical stress, making this material promising for use in microelectronics.^79^ A niccolite structured MOF, [(CH_3_CH_2_)_2_NH_2_][Fe^III^Fe^II^(HCOO)_6_], also demonstrated different dielectric constants, because of temperature induced phase transition, with a high‐dielectric state in the paraelastic phase, and a low‐one state in the ferroelastic phase, which is associated with statistical fluctuations of polar cations of diethylamine. The hysteresis for this process is in the temperature range between 227 and 240 K, while the values of the dielectric constants are 13 and 16, respectively.^80^

The ability to chemically tune the polarizability of MOFs, and developing film technology,^27^, ^28^ will support the creation new generation of field‐effect transistors, memristors, and microelectronic circuits based on porous MOFs as passive elements.^81^

### 3.2 Phase‐Change Effect

**Figure 5:**
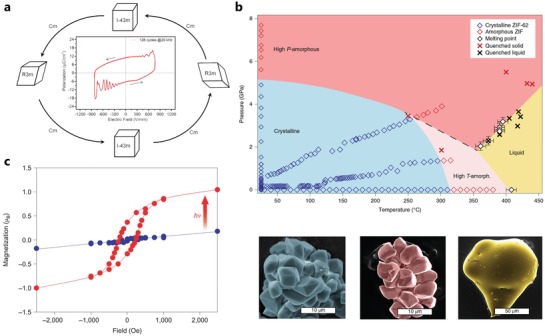
a) Reversible electrical tuning the crystal structure of model MOF. Reproduced with permission.^82^ Copyright 2019, American Association for the Advancement of Science. b) Pressure–temperature phase diagram for ZIF‐62: there are two amorphous phases (high‐*P*/high‐*T*) with high and low densities, a liquid phase, and crystalline field for the low pressure and low temperature condition. Reproduced with permission.^85^ Copyright 2019, Springer Nature. c) Light induced switching the magnetic properties of MOF: after irradiation by 473 nm light, a magnetic hysteresis loop with a coercive field of 240 Oe appeared. Reproduced permission.^96^ Copyright 2019, Springer Nature.

A phase transition describes stimulated reversible and nonreversible switching between two energy states favorable to a structure, and can be observed when stimuli as varied as pressure and temperature, as well as magnetic/electric fields, guest molecules or light, act on a material.^74^, ^82^ The structural changes (**Figure** **5**a) can provoke changes in the material's electrical, magnetic, dielectric, and optical properties.

The phase diagram of a well‐studied group of MOFs (ZIF) was recently established, showing several uncommon features. By increasing either the pressure or the temperature, crystalline to amorphous phase transitions could be observed.^83^, ^84^ However, while the pressure induced phase transition is displacive and therefore reversible,^84^ the temperature induced phase transition is reconstructive and irreversible (Figure 5b).^85^ A further increase in temperature leads to a transition to liquid state.^86^ Interestingly, the amorphous phases as well as the liquid phase maintain a significant level of porosity.^86^ While some consequences on both the chemical and processing properties^87^ have been observed, the implication of such behavior on the physical properties so far remains unclear. Another obvious example on temperature induced transition is 3D ZIF‐4, which undergoes an amorphous transformation at 300 °C, and a further irreversible recrystallization to the 3D ZIF‐zni state at 400 °C. This irreversible effect also changes its elastic constants.^88^ In any case, in ambient conditions, phase transitions are also observed under external magnetic or electric fields, light or guests with spectacular implications for the physical properties of MOFs.

3D [Cu_2_(dicarboxylate)_2_(amine)]*n* MOFs exhibit irreversible structural deformation when guest molecules are incorporated into their structure and then excluded.^89^ The penetration of gas through ZIF‐8 membranes can also be switched using an external electric field. This effect is explained by the structural transition of ZIF‐8 into polymorphs with a more rigid structure at 500 V mm^−1^.^82^ In addition, selective adsorption of CO was demonstrated for 3D MOFs with coordinated unsaturated iron (II) sites, based on the spin state transition of the iron (II) sites.^90^

Magnetic phase transitions have been investigated on a theoretical level for 2D MOFs with a range of metallic centers (Cr, Mn, Fe, Co, Ni),^91^ particularly, phase transition between homogeneous ferromagnetic and spin‐forming antiferromagnetic states. It was found that compounds with Mn exhibit strong d‐π hybridization, which leads to partially filled low‐spin levels, in contrast to compounds with transition metals, for which the Fermi energy is weakly split by spin due to weak d‐π hybridization and magnetic interaction.^91^ A switch between n (electron) and p (hole) type band structures has also been observed for 2D MOFs (M_3_C_12_S_12_ and M_3_C_12_O_12_, where M = Zn, Cd, Hg, Be, or Mg), due to tuneable deformation or electrostatic gating.^62^ In the case of chained MOFs such as NU‐901, one can observe electrochromic switching between yellow and deep blue hues by applied potential, due to a single‐electron redox reaction on their pyrene‐based linkers.^92^ A desired property of materials for data storage is the ability to switch magnetic domains. In this case, the 3D MOF, (CH_3_)_2_NH_2_Mn(HCOO)_3_, exhibits a perovskite architecture with ferroelectric domains, the magnetic states of which can be controlled by an electric field at temperatures below 180 K.^93^

At present, structures with reversible/nonreversible phase‐change effects are applied for triggers, switchers and other active elements for optical, magnetic, and microelectronic devices.^15^ The positive aspects for implementing MOFs in these fields are the efficient dynamic response of these complex structure to external stimuli. However, the diversity of energetically favorable states in a MOF structure, due to its complex conformational energy landscape,^94^ may complicate the reversible switching process.

### 3.3 Bistability

The fundamental ability of matter to have two equilibrium states in the same condition (bistability) underlies pivotal processes in life, from birth (cellular differentiation, apoptosis), to transportation (mechanical ratchets) and communication (memory devices, triggers). The latter raises a great technological challenge, with respect to the exponential growth in information that requires recording and processing. To address this, existing materials demonstrating bistability would have to permanently increase their record density, processing rate, and rewrite cycles. MOFs can be used as a new kind of active material for data storage. The basic principle for this application is their change in structure under electric, magnetic, mechanic, radiation, and other stimuli, which requires an additional coercive force for reversion to the initial structure.

Since bistability manifests itself as hysteresis (Figure 5c), it can be identified in MOFs as electrical, magnetic, mechanical, and electrochemical hysteresis. Cd(Imazethapyr)_2_, a 3D MOF, exhibits ferroelectric behavior and electrical hysteresis (with a remnent polarization, *P*_r_, of ≈0.006 µC cm^−2^, and coercive field, *E*_c_, of 0.9–1.1 kV cm^−1^), which is a typical feature for ferroelectrics.^95^

Magnetic hysteresis was observed in a 3D MOF based on Fe‐Nb assembly (Fe_2_[Nb(CN)_8_]·(4‐pyridinealdoxime)_8_·2H_2_O), which exhibited long‐range magnetic ordering of extended Fe^II^ (high‐spin) sites caused by photonic capture of an excited spin state. This MOF behaves like a spin‐crossover magnet, where strong mutual switching between photo‐emitted Fe^II^ and neighboring Nb^IV^ atoms works through C–N bridges. Magnetic phase transitions between low spin and high spin states have also been observed at 20 K with coercive fields of 240 Oe.^96^ The 3D niccolite MOF mentioned earlier, [(CH_3_CH_2_)_2_NH_2_][Fe^III^Fe^II^(HCOO)_6_], also appeared to demonstrate magnetic hysteresis regulated by a thermal and magnetic state at low temperatures,^80^ a phenomenon which is the response of the magnetization of Fe^II^ and Fe^III^ sublattices to external stimuli. This high bipolar reversible magnetization switching is different from the irreversible rotation of ferro/ferrimagnetic domains through the use of external magnetic fields.^80^

Mechanical hysteresis has been reported for some examples of 3D MOFs (e.g., rigid layers of Co/1,3,5‐benzenetricarboxylate which are connected to each other with flexible and spherical dipyridyl linkers). A special phase transition induces a new 2D structure without the loss of linkers, with the help of weak hydrogen bond acceptors. This phase transition can be reversed by heating the material in dimethylformamide.^97^

Light can also stimulate the transition between two structural states in MOFs with special photochromic molecules as ligands or guests.^98^, ^99^, ^100^, ^101^, ^102^, ^103^ The proton semiconductive hysteresis observed in the 3D MOF, Cu_2_(F_2_AzoBDC)_2_(dabco) (F_2_AzoBDC = (*E*)‐2‐((2,6‐difluorophenyl)diazenyl)terephthalic acid; dabco = 1,4‐Diazabicyclo[2.2.2]octane), is an indicator of such structural changes. This effect is achieved based on the MOF's azobenzene side groups, which can undergo light‐induced reversible isomerization between two *cis* and *trans* stable states. The photoisomerisation leads to modulation of the interaction between the MOF and guest molecules, allowing switching between states with increased (*trans*) and decreased (*cis*) proton conductivity.^98^ In general, this photoisomerisation process leads to conformation changes of the ligand, and modulates the electronic structure of MOFs and, therefore, their chemical,^98^, ^99^, ^100^, ^101^, ^102^ optical,^103^ and electrical^104^ properties. Also intriguing is the fact that the symmetry group of MOFs can be changed upon photoisomerisation,^102^ as this is especially important for optical data storage. However, problems still remain regarding the thermal relaxation for some compounds and the *trans*–*cis* transition rates in MOFs. Experimental results indicate that these materials demonstrate slow photoisomerisation (from minutes to hours), while other complicated molecules have demonstrated very fast photoisomerisation (up to picoseconds).^105^

Finally, electrochemical hysteresis was observed in a Fe_2_(BDP)_3_ 3D MOF with K^+^ ions as a guest, (K*_x_*Fe_2_(BDP)_3_, BDP^2−^ = 1,4‐benzenedipyrazolate). Slow scan cyclic voltammetry highlighted reversible conversion between the low spin iron (II) state at *x* = 0, and low and high spin iron (III) states at *x* = 1.1.^106^ Another 3D MOF, CoNDI‐py‐2 (NDI‐py = *N*,*N*′‐bis(4‐pyridyl)‐1,4,5,8‐naphthalene diimide) in MeCN, also exhibited similar electrochemical hysteresis. The voltammograms showed signs of naphthalene diimide reduction pairs at E_1/2_ = −0.79 and −1.25 V versus Ag/AgNO_3_, and bonding with the Co(II)/Co(I) pair at −0.79 V.^23^

Metal–organic frameworks demonstrating bistability are important materials for future memory devices such as memristors and random access memory modules.^28^, ^107^, ^108^, ^109^ However, there are still issues hindering their real‐world application, as an increase to the switching rate to THz and higher is required, and minimization of the area of tuneable MOFs needs to be addressed.

### 3.4 Spin Qubits

**Figure 6:**
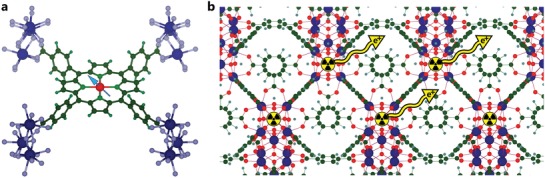
a) Illustration of MOF structure with a central paramagnetic ion as a clock qubit. The structure was extracted from CIF file.^110^ b) MOF structure with positron‐emitting isotopes. The structure was extracted from the CIF file.^128^

A qubit is the smallest physical element for storing information in quantum computers. A spin qubit records information based on the orientation of spins in coherent superposition. Very recently, MOFs have become objects of research for the implementation of this concept. An array of clocklike qubits was created within the framework of an organometallic structure (**Figure** **6**a). Studies have confirmed a clocklike transition for cobalt (II) spins in a 2D [(TCPP)Co_0.07_Zn_0.93_]_3_[Zr_6_O_4_(OH)_4_(H_2_O)_6_]_2_ (TCPP = 5,10,15,20‐tetrakis(carboxyphenyl)porphyrin) framework. The lifetime of the clocklike spin qubits was 14 µs, despite the local abundant nuclear rotation of the spins.^110^ It was also shown that, as a node in the 2D MOF, [{CuTCPP}Zn_2_(H_2_O)_2_], an isolated molecule, retains quantum coherence and is a potential spin qubit. Phase coherence in this material is independent of temperature up to 30 K. The qubit lifetime is 2.24 µs at 6 K, and decreases to 0.85 µs at 60 K, possibly due to thermal processes associated with relaxation of the spin lattice and softening of the frozen solution.^111^

Generally, spin qubits can be used for quantum processing or as data storage elements of the future because they are subject to quantum physics laws and operate at the minimal scale single atoms.

## 4 MOFs in Particle Physics

Based on specific metal clusters, porous crystals can be considered to be one of the main elements for applications in particle physics, from positronium emission, to radiation detectors and radioactive probes. This field is in its infancy, with pioneer works only being published since 2006, and possesses great potential for further development. Here, we discuss state‐of‐the‐art MOFs for particle physics in the terms of their structure.

### 4.1 Long‐Lived Positronium

Positronium (Ps), also known as the lightest atom in nature, is a bound state of an electron and a positron (β^+^). Ps atoms have prompted substantial interest for both fundamental and applied physics.^112^ Ps gas can be produced by the interaction of β^+^ beams with gases or solids. The short lifetime of Ps atoms is one of the main problems hindering their use. In conventional solids they can be trapped by crystal defects and annihilated, producing two γ quanta with 511 keV of energy, each. In contrast, in the highly porous MOF medium, newly formed Ps atoms immediately move into crystal voids, drastically increasing their lifetime.^113^ Studies have shown that Ps exists in a number of MOFs (ZnO_4_(FMA)_3_, MOF‐5, ZIF‐8, IRMOF‐8, and IRMOF‐20), in a delocalized Bloch state that protects them from being trapped by some crystal irregularities.^113^, ^114^, ^115^

### 4.2 Radiation Detection

The undeniable danger of various types of ionizing radiation lies in the fact that their actions are not perceptible until the onset of adverse effects. Nevertheless, under strictly controlled conditions, radioactive drugs, γ rays, and proton and neutron beams can be used for medical diagnostics and treatment. Highly active radiation sources and fuels are also widely used in industry and nuclear power. Therefore, it is important to develop new materials for the detection and control of ionizing radiation.

The scintillation technique is one of many principles of radiation detection, and is based on the radioluminescence effect observed in some materials (alkali halides, silicates, plastics, and organic crystals). MOFs have occupied a niche as scintillating materials for over a decade.^116^ In ref. 116, Doty et al. exposed Zn_4_O(SDC)_3_ (SDC is stilbene dicarboxylate) and Zn_3_(SDC)_3_(DMF)_2_ (DMF is *N*,*N*‐dimethylformamide) to both a 3 MeV proton beam and an alpha particle source, and demonstrated that the visible light output from these MOFs is comparable to those of commercially available scintillators. In addition, their radiation resistance is much higher than that of the anthracene crystal standard. Recently, Mathis et al. synthesized and characterized scintillation performance (using 2.5 MeV protons) of stilbene‐ and anthracene‐based lanthanide MOFs (Eu^3+^, Tb^3+^, Er^3+^, Tm^3+^).^117^, ^118^ Lu et al. used Pb^2+^ naphthalene‐based MOFs for absorption and detection of X‐rays. These studies support the assertion that MOFs are one of the key functional materials in physics.^119^

### 4.3 Radioactive MOFs

A variety of MOFs can be produced based on actinides and other radioactive isotopes.^120^, ^121^, ^122^, ^123^ Recently, Andreo et al. synthesized a Th‐based MOF containing 2,6‐naphthalendicarboxylic acid as a scintillating ligand,^124^ with the resulting crystal demonstrating autoluminescence properties due to radioactive Th decay. To date, MOFs have been successfully used for medical applications and sensing,^11^, ^12^, ^125^, ^126^ particularly in positron emission tomography (PET), which is based on simultaneous 180° detection of two 511 keV γ quanta appearing after β^+^ annihilation.^127^ Metabolites doped with β^+^‐radioactive isotopes like ^18^F, ^11^C, ^13^N, ^15^O, act as molecular probes for the location of tumors, and the study of artery diseases and other pathologies. Chen et al. showed that the ^89^Zr‐UiO‐66 MOF (Figure 6b) functionalized with pyrene‐derived polyethylene glycol and conjugated with a peptide ligand could be used for targeting breast tumors.^128^

## 5 Artificial MOF Structures

Finally, metal–organic frameworks represent a clear example of hierarchical solids containing structural elements such as ion clusters and ligands, which themselves have structure.^129^ Such structural hierarchy is a solution for observation of the unconventional structure‐related physical properties of modern materials.^130^, ^131^ In light of this, following from reticular synthesis and self‐assembly, one can increase the level of hierarchy, yielding new MOF super‐ and meso‐structures with structure‐related optical effects. Moreover, considering the hierarchy of MOFs, one can compare them to other mechanisms demonstrating extraordinary behavior, such as artificial man‐made mechanical metamaterials (MM) and origami.^130^, ^132^ Here we discuss the perspectives of MOFs for fabricating new kinds of optical superstructures and mechanical metamaterials at the molecular level.

### 5.1 Optical Superstructures

Hierarchically complex structures exhibit special properties that are not observed with the base materials used to assemble these structures. This synergistic effect is often applied in architecture and photonics, where there are examples of many natural and artificial hierarchical structures, such as photonic crystals and metasurfaces, for controlling light at the nanoscale. The characteristic periods of such structures are commensurate to resonant optical wavelengths, enabling complex interference and, accordingly, control of light propagation. Over the past decade, MOFs have proven to be a potential building block for photonic structures with enhanced functionality.

**Figure 7:**
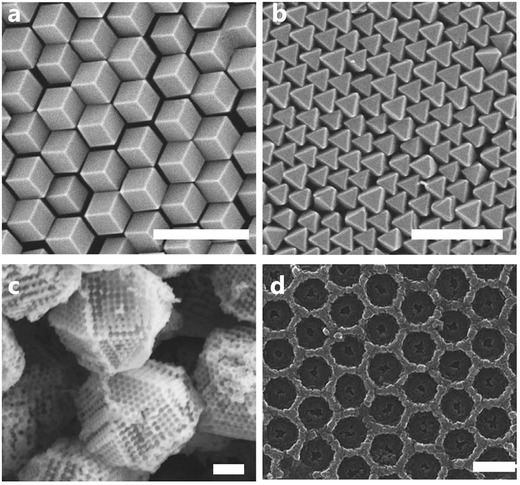
a,b) Self‐assembed 2D MOF photonic structures as tunable optical sensors.^135^ Scale bar, 1 µm. c,d) MOF photonic structures in the form of inverted opals.^134^, ^138^ Scale bars, 1 µm. a,b) Reproduced with permission.^135^ Copyright 2019, Springer Nature. c) Reproduced with permission.^138^ Copyright 2019, American Association for the Advancement of Science. d) Reproduced with permission.^134^ Copyright 2019, Springer Nature.

The construction of super‐ and meso‐structured MOFs occurs mainly by self‐assembly, and they can be ideally located on a range of substrates in different ways,^133^, ^134^ while controlling their size, shape, and functionalities.^27^, ^92^ A clear example of this is assembly of polyhedral particles of TRD ZIF‐8 crystals, synthesized with good monodispersity, uniformity, and colloidal stability. Such particles are then spontaneously assembled into millimeter‐ordered structures, creating photonic crystals, allowing them to dynamically change the wavelength of reflected light under gas sorption (**Figure** **7**a,b).^135^ The study of the use of spherical MOF colloidal crystals as matrices has also highlighted the possibilities for recognition of guest molecules with unique signaling. The unique integrated gap in such spheres provides a good way to manipulate the photophysical and photochemical behavior of the guest molecules inside the MOF superstructure, when it is used as a photonic structure.^136^ Another example is with MOF‐based diffraction gratings:^137^ MIL‐101(Cr), obtained by applying a dip‐coating process on a range of substrates, opens up the production of low thickness superstructures with ordered cracks, depending on the evaporation front and the rate of substrate removal from the solvent.

Self‐assembly strategies, the development of thin film technology, and a dynamic structural response enable MOF superstructures to be applied for optical sensors based on photonic crystals,^138^, ^139^, ^140^, ^141^ electro‐optical devices,^16^ light switchers,^142^ and adaptive reflectors.^135^, ^137^ Moreover, as single 100–300 nm nanoparticles,^143^ the structures are promising for nanometer‐scale light manipulation, due to the generation of specific Mie‐type optical resonances (so‐called all‐dielectric nanophotonics).^144^, ^145^ However, the current problem to be solved is maintaining the consistency of complex MOF structures under actual operating conditions, focusing on mechanical stress, humidity, and degradation in air.

### 5.2 Mechanical Metamaterials

**Figure 8:**
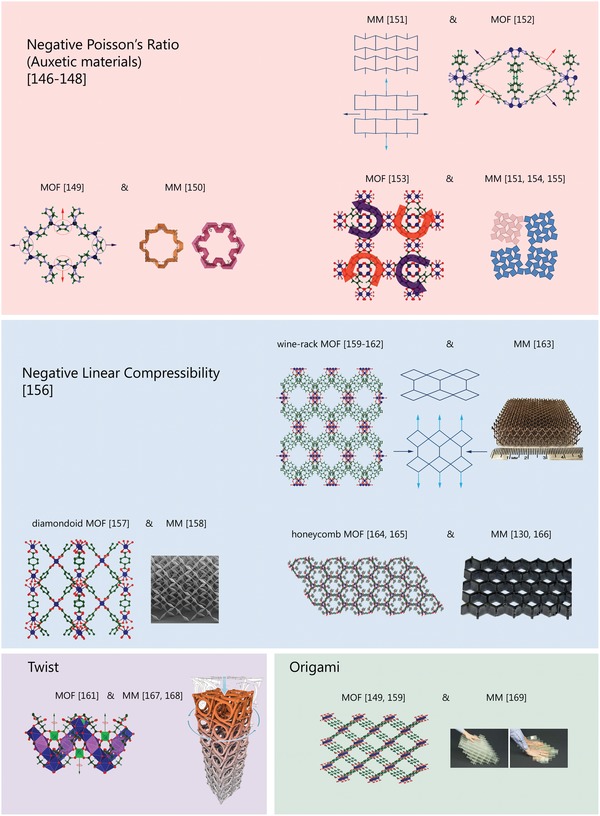
Comparison of the structures of MOFs and metamaterials (MM) having similar unconventional mechanical properties as negative Poisson's ratio, negative linear compressibility, twisting under the pressure, and origami behavior that means an ability to lay the structure in one direction and inability in another (navy arrows indicate the stimuli, red and blue ones correspond to structural response). The MOF structures were extracted from CIF files.^149^, ^152^, ^153^, ^157^, ^159^, ^160^, ^161^, ^164^ Reproduced with permission.^150^ Copyright 2017, Springer Nature. Reproduced with permission.^154^ Copyright 2015, Springer Nature. Reproduced with permission.^169^ Copyright 2016, Springer Nature. Reproduced with permission.^163^ Copyright 2011, American Association for the Advancement of Science. Reproduced with permission.^166^ Copyright 2016, American Association for the Advancement of Science. Reproduced with permission.^168^ Copyright 2017, American Association for the Advancement of Science. Reproduced with permission.^158^ Copyright 2012, American Institute of Physics.

In terms of molecular architecture, metal–organic frameworks are hierarchical structures based on building blocks with different topologies, linked together by different weak and strong bonds (Figure 3). This arrangement facilitates complicated mechanical behavior under external stimuli, and the literature is full of articles devoted to analysis of the unconventional mechanics of MOFs. An intriguing finding is that the hierarchical MOF structure itself is similar to a metamaterial (see **Figure** **8** illustrating results from refs. 146, 147, 148, 149, 150, 151, 152, 153, 154, 155, 156, 157, 158, 159, 160, 161, 162, 163, 164, 165, 166, 167, 168, 169), which is characterized by the different interactions (mechanical, electromagnetic) between the central element, a meta atom of a specific shape and size, with its neighbors.^170^ Metamaterials also demonstrate unconventional mechanical behavior. Based on this behavior, we compared these structures to MOFs, and found a direct similarity in their structure‐related properties.^171^ This similarity allows MOFs to be considered a molecular scale mechanical metamaterial.

Figure 8 represents different MOF structures (e.g., wine‐racks, diamondoids, and honeycombs) which can twist under pressure, and exhibit negative Poisson's ratio, negative linear compressibility, and origami behavior, because the mobility of the frame is determined by their specific soft structure. For instance, quantum‐mechanical calculations of the elastic constants for MIL‐53(Al), MIL‐53(Ga), MIL‐47, DMOF‐1, and DMOF‐1 highlighted highly anisotropic behavior, with some directions demonstrating low shear and Young's modulus. This is explained by the fact that inorganic chains correspond to more rigid directions, whereas soft directions belong to the deformation vibration modes.^159^ This effect can be considered to be origami behavior.^170^, ^172^ Another example is MOF, [Zn(L)_2_(OH)_2_]*n*∙Guest (where L is 4‐(1*H*‐naphtho[2,3‐*d*]imidazol‐1‐yl)benzoate, and guest is water or methanol), which exhibits a negative compressibility area that can be customized by replacing/exchanging guest molecules.^173^ When heated, such materials are compressed in one direction with negative linear strain. In the 3D MOF, [Ag(ethylenediamine)]NO_3_, this direction coincides with the strongest direction of positive thermal expansion, due to the relationship of temperature with pressure, and the conjugation of compressibility with expansiveness.^174^ Network‐based metamaterials also demonstrate such negative compressibility.^175^

Studies of the thermomechanical and viscoelastic properties of the HKUST‐1 MOF revealed a decrease in Young's modulus during compression, as well as a decrease in hardness with temperature increasing from 25 to 100 °C. This means that thermally induced softening is more important than thermally induced increases in density.^176^

Studies of the mechanical properties and energy absorption in single crystals of four isostructural samples belonging to the UiO type of MOF highlighted plasticity and endothermicity during deformation. These characteristics are associated with their unique property of absorption and scattering of mechanical shock waves from the outside. At stresses higher than 2 GPa, energy absorption reaches 3–4 kJ per gram (for reference, about 4 kJ of energy per gram is released on the explosion of TNT, trinitrotoluol equivalent).^177^ Studies of the elastic constants of different types of MOFs (e.g., ZIFs, Cu_1.5_(H_2_O)(O_3_PCH_2_CO_2_), Ce(C_2_O_4_)(HCO_2_), Zn_3_(PO_4_)(O_2_CCH_2_PO_3_)(H_2_O), MOF‐5, IRMOFs, LiB(Im)4) also revealed changes to the bulk modulus, the Young's modulus, and the Poisson ratio.^38^ Such MOFs can be used as mechanical elements in thermoelectronic and nanospring devices, flexible electronics, and for damping and dissipating external voltage. Moreover, as opposed to conventional materials, the presence of structural defects improves the mechanical response of MOFs to a certain extent,^178^ such that the “intrinsic defects” term requires reconsideration when applied to MOFs.^179^

## 6 Conclusion

**Figure 9:**
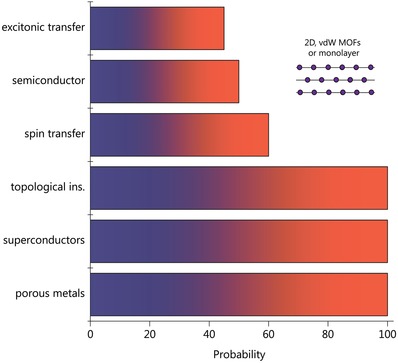
The family of 2D MOFs demonstrating different electronic properties. The statistical analysis of structure‐related properties was performed in Scopus. Topological states, superconductivity, and metallic conductivity are demonstrated only by 2D/layered MOFs.

Until recently, the development of MOF technology has been motivated by their unprecedented sorption, filtration, and capacitance properties, which suggested that they could break new ground in the chemical industry.^1^, ^7^ The main challenge this development faced was increasing the volume and functionality of nanometer‐scale pores. Now, MOFs are entering new fields of science, in optics, communications, particle physics, and mechanics, as researchers look beyond their porosity. By focusing attention on the combination of crystallinity, softness, organic–inorganic nature, and complex hierarchy, inherent to MOFs, one can observe intriguing physical properties suitable for energy transfer, data storage, radiation detection, and particle emission to nonlinear mechanics (Figure 2). Moreover, from the physical point of view, 2D MOFs are also promising for their metallic conductivity, superconductivity, topological insulators, and other unconventional electronic effects (**Figure** **9**).

Looking into the future, we believe that further research in physics of metal–organic frameworks will focus on classical and quantum data storage with higher rates and record densities,^107^, ^108^, ^109^, ^110^ lasing within the visible^53^ and THz ranges, spintronics,^63^ excitonics,^46^, ^52^ valeytronics, and absorbing light, microwaves, and shock waves.^177^, ^180^ With these prospects at hand, the new world of industry may be opened for the porous crystals.

## Conflict of Interest

The authors declare no conflict of interest.

## References

[1] H. Furukawa , K. E. Cordova , M. O'Keeffe , O. M. Yaghi , Science 2013, 341, 1230444. 23990564 10.1126/science.1230444

[2] Y. Kinoshita , I. Matsubara , T. Higuchi , Y. Saito , Bull. Chem. Soc. Jpn. 1959, 32, 1221.

[3] H. Li , M. Eddaoudi , M. O'Keeffe , O. M. Yaghi , Nature 1999, 402, 276.

[4] W. J. Rieter , K. M. L. Taylor , H. An , W. Lin , W. Lin , J. Am. Chem. Soc. 2006, 128, 9024. 16834362 10.1021/ja0627444 PMC2556368

[5] B. Liu , H. Shioyama , T. Akita , Q. Xu , J. Am. Chem. Soc. 2008, 130, 5390. 18376833 10.1021/ja7106146

[6] Y. Wu , F. Li , W. Zhu , J. Cui , C. Tao , C. Lin , P. M. Hannam , G. Li , Angew. Chem. 2011, 123, 12726; 10.1002/anie.201104597 22006864

[7] H. Furukawa , U. Müller , O. M. Yaghi , Angew. Chem. 2015, 127, 3480; 10.1002/anie.201410252 25586609

[8] A. Pulido , L. Chen , T. Kaczorowski , D. Holden , M. A. Little , S. Y. Chong , B. J. Slater , D. P. McMahon , B. Bonillo , C. J. Stackhouse , A. Stephenson , C. M. Kane , R. Clowes , T. Hasell , A. I. Cooper , G. M. Day , Nature 2017, 543, 657. 28329756 10.1038/nature21419 PMC5458805

[9] S. Horike , S. Shimomura , S. Kitagawa , Nat. Chem. 2009, 1, 695. 21124356 10.1038/nchem.444

[10] L. R. Mingabudinova , V. V. Vinogradov , V. A. Milichko , E. Hey‐Hawkins , A. V. Vinogradov , Chem. Soc. Rev. 2016, 45, 5408. 27711673 10.1039/c6cs00395h

[11] K. Lu , T. Aung , N. Guo , R. Weichselbaum , W. Lin , Adv. Mater. 2018, 30, 1707634. 10.1002/adma.201707634 PMC6586248 29971835

[12] A. C. McKinlay , R. E. Morris , P. Horcajada , G. Férey , R. Gref , P. Couvreur , C. Serre , Angew. Chem. 2010, 122, 6400; 10.1002/anie.201000048 20652915

[13] J. Heine , K. Müller‐Buschbaum , Chem. Soc. Rev. 2013, 42, 9232. 24077361 10.1039/c3cs60232j

[14] P. Ramaswamy , N. E. Wong , G. K. H. Shimizu , Chem. Soc. Rev. 2014, 43, 5913. 24733639 10.1039/c4cs00093e

[15] A. Walsh , K. T. Butler , C. H. Hendon , MRS Bull. 2016, 41, 870.

[16] W.‐H. Li , K. Ding , H.‐R. Tian , M.‐S. Yao , B. Nath , W.‐H. Deng , Y. Wang , G. Xu , Adv. Funct. Mater. 2017, 27, 1702067.

[17] L. Sun , C. H. Hendon , S. S. Park , Y. Tulchinsky , R. Wan , F. Wang , A. Walsh , M. Dincă , Chem. Sci. 2017, 8, 4450. 28616149 10.1039/c7sc00647k PMC5452916

[18] M. G. Campbell , D. Sheberla , S. F. Liu , T. M. Swager , M. Dincă , Angew. Chem. 2015, 127, 4423; 10.1002/anie.201411854 25678397

[19] L.‐P. Tang , L.‐M. Tang , H. Geng , Y.‐P. Yi , Z. Wei , K.‐Q. Chen , H.‐X. Deng , Appl. Phys. Lett. 2018, 112, 012101.

[20] D. Sheberla , L. Sun , M. A. Blood‐Forsythe , S. Er , C. R. Wade , C. K. Brozek , A. Aspuru‐Guzik , M. Dincă , J. Am. Chem. Soc. 2014, 136, 8859. 24750124 10.1021/ja502765n

[21] X. Huang , P. Sheng , Z. Tu , F. Zhang , J. Wang , H. Geng , Y. Zou , C.‐A. Di , Y. Yi , Y. Sun , W. Xu , D. Zhu , Nat. Commun. 2015, 6, 7408. 26074272 10.1038/ncomms8408 PMC4490364

[22] L. S. Xie , L. Sun , R. Wan , S. S. Park , J. A. DeGayner , C. H. Hendon , M. Dincă , J. Am. Chem. Soc. 2018, 140, 7411. 29807428 10.1021/jacs.8b03604

[23] E. Castaldelli , K. D. G. I. Jayawardena , D. C. Cox , G. J. Clarkson , R. I. Walton , L. Le‐Quang , J. Chauvin , S. R. P. Silva , G. J.‐F. Demets , Nat. Commun. 2017, 8, 2139. 29247248 10.1038/s41467-017-02215-7 PMC5732256

[24] R. Dong , P. Han , H. Arora , M. Ballabio , M. Karakus , Z. Zhang , C. Shekhar , P. Adler , P. St. Petkov , A. Erbe , S. C. B. Mannsfeld , C. Felser , T. Heine , M. Bonn , X. Feng , E. Cánovas , Nat. Mater. 2018, 17, 1027. 30323335 10.1038/s41563-018-0189-z

[25] J. Park , M. Lee , D. Feng , Z. Huang , A. C. Hinckley , A. Yakovenko , X. Zou , Y. Cui , Z. Bao , J. Am. Chem. Soc. 2018, 140, 10315. 30041519 10.1021/jacs.8b06020

[26] C. Wang , X. Liu , N. K. Demir , J. P. Chen , K. Li , Chem. Soc. Rev. 2016, 45, 5107. 27406473 10.1039/c6cs00362a

[27] P. Falcaro , R. Ricco , C. M. Doherty , K. Liang , A. J. Hill , M. J. Styles , Chem. Soc. Rev. 2014, 43, 5513. 24802634 10.1039/c4cs00089g

[28] L. Heinke , C. Wöll , Adv. Mater. 2019, 31, 1806324. 10.1002/adma.201806324 30701602

[29] M. Usman , S. Mendiratta , K.‐L. Lu , Adv. Mater. 2017, 29, 1605071. 10.1002/adma.201605071 27859732

[30] A. J. Clough , J. M. Skelton , C. A. Downes , A. A. de la Rosa , J. W. Yoo , A. Walsh , B. C. Melot , S. C. Marinescu , J. Am. Chem. Soc. 2017, 139, 10863. 28704606 10.1021/jacs.7b05742

[31] T. Kambe , R. Sakamoto , T. Kusamoto , T. Pal , N. Fukui , K. Hoshiko , T. Shimojima , Z. Wang , T. Hirahara , K. Ishizaka , S. Hasegawa , F. Liu , H. Nishihara , J. Am. Chem. Soc. 2014, 136, 14357. 25251306 10.1021/ja507619d

[32] L. Sun , M. G. Campbell , M. Dincă , Angew. Chem. 2016, 128, 3628; 10.1002/anie.201506219 26749063

[33] J.‐H. Dou , L. Sun , Y. Ge , W. Li , C. H. Hendon , J. Li , S. Gul , J. Yano , E. A. Stach , M. Dincă , J. Am. Chem. Soc. 2017, 139, 13608. 28910095 10.1021/jacs.7b07234

[34] Y. He , E. D. Cubuk , M. D. Allendorf , E. J. Reed , J. Phys. Chem. Lett. 2018, 9, 4562. 30052453 10.1021/acs.jpclett.8b01707

[35] F. Li , X. Zhang , X. Liu , M. Zhao , ACS Appl. Mater. Interfaces 2018, 10, 15012. 29658262 10.1021/acsami.8b00942

[36] O. M. Yaghi , M. O'Keeffe , N. W. Ockwig , H. K. Chae , M. Eddaoudi , J. Kim , Nature 2003, 423, 705. 12802325 10.1038/nature01650

[37] M. M. Labes , P. Love , L. F. Nichols , Chem. Rev. 1979, 79, 1.

[38] X. Blase , E. Bustarret , C. Chapelier , T. Klein , C. Marcenat , Nat. Mater. 2009, 8, 375. 19387452 10.1038/nmat2425

[39] X. Zhang , Y. Zhou , B. Cui , M. Zhao , F. Liu , Nano Lett. 2017, 17, 6166. 28898086 10.1021/acs.nanolett.7b02795

[40] Y. Ye , Z. J. Wong , X. Lu , X. Ni , H. Zhu , X. Chen , Y. Wang , X. Zhang , Nat. Photonics 2015, 9, 733.

[41] J. R. Schaibley , H. Yu , G. Clark , P. Rivera , J. S. Ross , K. L. Seyler , W. Yao , X. Xu , Nat. Rev. Mater. 2016, 1, 16055.

[42] V. A. Milichko , A. S. Shalin , I. S. Mukhin , A. E. Kovrov , A. A. Krasilin , A. V. Vinogradov , P. A. Belov , C. R. Simovski , Physics‐Uspekhi 2016, 59, 727.

[43] A. V. Vinogradov , V. A. Milichko , H. Zaake‐Hertling , A. Aleksovska , S. Gruschinski , S. Schmorl , B. Kersting , E. M. Zolnhofer , J. Sutter , K. Meyer , P. Lönnecke , E. Hey‐Hawkins , Dalton Trans. 2016, 45, 7244. 26906040 10.1039/c6dt00390g

[44] L. Cao , Z. Lin , W. Shi , Z. Wang , C. Zhang , X. Hu , C. Wang , W. Lin , J. Am. Chem. Soc. 2017, 139, 7020. 28467852 10.1021/jacs.7b02470

[45] C. A. Kent , D. Liu , L. Ma , J. M. Papanikolas , T. J. Meyer , W. Lin , J. Am. Chem. Soc. 2011, 133, 12940. 21776996 10.1021/ja204214t

[46] H.‐J. Son , S. Jin , S. Patwardhan , S. J. Wezenberg , N. C. Jeong , M. So , C. E. Wilmer , A. A. Sarjeant , G. C. Schatz , R. Q. Snurr , O. K. Farha , G. P. Wiederrecht , J. T. Hupp , J. Am. Chem. Soc. 2013, 135, 862. 23249338 10.1021/ja310596a

[47] Q. Zhang , C. Zhang , L. Cao , Z. Wang , B. An , Z. Lin , R. Huang , Z. Zhang , C. Wang , W. Lin , J. Am. Chem. Soc. 2016, 138, 5308. 27016183 10.1021/jacs.6b01345

[48] J. Lin , X. Hu , P. Zhang , A. van Rynbach , D. N. Beratan , C. A. Kent , B. P. Mehl , J. M. Papanikolas , T. J. Meyer , W. Lin , S. S. Skourtis , M. Constantinou , J. Phys. Chem. C 2013, 117, 22250.

[49] P. Mahato , A. Monguzzi , N. Yanai , T. Yamada , N. Kimizuka , Nat. Mater. 2015, 14, 924. 26237125 10.1038/nmat4366

[50] C. Y. Lee , O. K. Farha , B. J. Hong , A. A. Sarjeant , S. B. T. Nguyen , J. T. Hupp , J. Am. Chem. Soc. 2011, 133, 15858. 21916479 10.1021/ja206029a

[51] W.‐M. Liao , J.‐H. Zhang , S.‐Y. Yin , H. Lin , X. Zhang , J. Wang , H.‐P. Wang , K. Wu , Z. Wang , Y.‐N. Fan , M. Pan , C.‐Y. Su , Nat. Commun. 2018, 9, 2401. 29921871 10.1038/s41467-018-04833-1 PMC6008449

[52] V. A. Milichko , S. V. Makarov , A. V. Yulin , A. V. Vinogradov , A. A. Krasilin , E. Ushakova , V. P. Dzyuba , E. Hey‐Hawkins , E. A. Pidko , P. A. Belov , Adv. Mater. 2017, 29, 1606034. 10.1002/adma.201606034 28112457

[53] R. Medishetty , V. Nalla , L. Nemec , S. Henke , D. Mayer , H. Sun , K. Reuter , R. A. Fischer , Adv. Mater. 2017, 29, 1605637. 10.1002/adma.201605637 28218491

[54] J. Yu , J. H. Park , A. V. Wyk , G. Rumbles , P. Deria , J. Am. Chem. Soc. 2018, 140, 10488. 30040404 10.1021/jacs.8b04980

[55] X. Liu , S. Akerboom , M. de Jong , I. Mutikainen , S. Tanase , A. Meijerink , E. Bouwman , Inorg. Chem. 2015, 54, 11323. 26599972 10.1021/acs.inorgchem.5b01924

[56] D. Unuchek , A. Ciarrocchi , A. Avsar , K. Watanabe , T. Taniguchi , A. Kis , Nature 2018, 560, 340. 30046107 10.1038/s41586-018-0357-y

[57] K. S. Novoselov , A. Mishchenko , A. Carvalho , A. H. Castro Neto , Science 2016, 353, aac9439. 27471306 10.1126/science.aac9439

[58] J. D. Plumhof , T. Stöferle , L. Mai , U. Scherf , R. F. Mahrt , Nat. Mater. 2014, 13, 247. 24317189 10.1038/nmat3825

[59] T. Byrnes , N. Y. Kim , Y. Yamamoto , Nat. Phys. 2014, 10, 803.

[60] V. A. Milichko , E. V. Khramenkova , V. P. Dzyuba , E. A. Pidko , Adv. Mater. 2017, 29, 1705261. 10.1002/adma.201705261 29239521

[61] S. A. Wolf , D. D. Awschalom , R. A. Buhrman , J. M. Daughton , S. von Molnár , M. L. Roukes , A. Y. Chtchelkanova , D. M. Treger , Science 2001, 294, 1488. 11711666 10.1126/science.1065389

[62] M. Wu , Z. Wang , J. Liu , W. Li , H. Fu , L. Sun , X. Liu , M. Pan , H. Weng , M. Dincă , 2D Mater. 2017, 4, 015015.

[63] M. G. Yamada , H. Fujita , M. Oshikawa , Phys. Rev. Lett. 2017, 119, 057202. 28949730 10.1103/PhysRevLett.119.057202

[64] M. G. Yamada , V. Dwivedi , M. Hermanns , Phys. Rev. B 2017, 96, 155107.

[65] B. Yan , C. Felser , Annu. Rev. Condens. Matter Phys. 2017, 8, 337.

[66] L. Lu , J. D. Joannopoulos , M. Soljačić , Nat. Photonics 2014, 8, 821.

[67] Z. F. Wang , N. Su , F. Liu , Nano Lett. 2013, 13, 2842. 23678979 10.1021/nl401147u

[68] L. Z. Zhang , Z. F. Wang , B. Huang , B. Cui , Z. Wang , S. X. Du , H.‐J. Gao , F. Liu , Nano Lett. 2016, 16, 2072. 26866565 10.1021/acs.nanolett.6b00110

[69] X. Ni , W. Jiang , H. Huang , K.‐H. Jin , F. Liu , Nanoscale 2018, 10, 11901. 29897371 10.1039/c8nr02651c

[70] N. Su , W. Jiang , Z. Wang , F. Liu , Appl. Phys. Lett. 2018, 112, 033301.

[71] M. G. Yamada , T. Soejima , N. Tsuji , D. Hirai , M. Dincă , H. Aoki , Phys. Rev. B 2016, 94, 081102(R).

[72] O. Sato , Nat. Chem. 2016, 8, 644. 27325090 10.1038/nchem.2547

[73] H. Deng , M. A. Olson , J. F. Stoddart , O. M. Yaghi , Nat. Chem. 2010, 2, 439. 20489710 10.1038/nchem.654

[74] F.‐X. Coudert , Chem. Mater. 2015, 27, 1905.

[75] D. Shamiryan , T. Abell , F. Iacopi , K. Maex , Mater. Today 2004, 7, 34.

[76] G. Bersuker , P. Zeitzoff , G. Brown , H. R. Huff , Mater. Today 2004, 7, 26.

[77] M. R. Ryder , Z. Zeng , K. Titov , Y. Sun , E. M. Mahdi , I. Flyagina , T. D. Bennett , B. Civalleri , C. S. Kelley , M. D. Frogley , G. Cinque , J.‐C. Tan , J. Phys. Chem. Lett. 2018, 9, 2678. 29724101 10.1021/acs.jpclett.8b00799

[78] S. Eslava , L. Zhang , S. Esconjauregui , J. Yang , K. Vanstreels , M. R. Baklanov , E. Saiz , Chem. Mater. 2013, 25, 27.

[79] W.‐J. Li , J. Liu , Z.‐H. Sun , T.‐F. Liu , J. Lü , S.‐Y. Gao , C. He , R. Cao , J.‐H. Luo , Nat. Commun. 2016, 7, 11830. 27282348 10.1038/ncomms11830 PMC4906389

[80] J.‐P. Zhao , J. Xu , S.‐D. Han , Q.‐L. Wang , X.‐H. Bu , Adv. Mater. 2017, 29, 1606966.

[81] N. C. Burtch , J. Heinen , T. D. Bennett , D. Dubbeldam , M. D. Allendorf , Adv. Mater. 2018, 30, 1704124. 10.1002/adma.201704124 29149545

[82] A. Knebel , B. Geppert , K. Volgmann , D. I. Kolokolov , A. G. Stepanov , J. Twiefel , P. Heitjans , D. Volkmer , J. Caro , Science 2017, 358, 347. 29051376 10.1126/science.aal2456

[83] T. D. Bennett , Y. Yue , P. Li , A. Qiao , H. Tao , N. G. Greaves , T. Richards , G. I. Lampronti , S. A. T. Redfern , F. Blanc , O. K. Farha , J. T. Hupp , A. K. Cheetham , D. A. Keen , J. Am. Chem. Soc. 2016, 138, 3484. 26885940 10.1021/jacs.5b13220

[84] T. D. Bennett , P. Simoncic , S. A. Moggach , F. Gozzo , P. Macchi , D. A. Keen , J.‐C. Tana , A. K. Cheetham , Chem. Commun. 2011, 47, 7983. 10.1039/c1cc11985k 21681315

[85] R. N. Widmer , G. I. Lampronti , S. Anzellini , R. Gaillac , S. Farsang , C. Zhou , A. M. Belenguer , C. W. Wilson , H. Palmer , A. K. Kleppe , M. T. Wharmby , X. Yu , S. M. Cohen , S. G. Telfer , S. A. T. Redfern , F.‐X. Coudert , S. G. MacLeod , T. D. Bennett , Nat. Mater. 2019, 18, 370. 30886398 10.1038/s41563-019-0317-4

[86] R. Gaillac , P. Pullumbi , K. A. Beyer , K. W. Chapman , D. A. Keen , T. D. Bennett , F.‐X. Coudert , Nat. Mater. 2017, 16, 1149. 29035353 10.1038/nmat4998

[87] T. D. Bennett , J.‐C. Tan , Y. Yue , E. Baxter , C. Ducati , N. J. Terrill , H. H. M. Yeung , Z. Zhou , W. Chen , S. Henke , A. K. Cheetham , G. N. Greaves , Nat. Commun. 2015, 6, 8079. 26314784 10.1038/ncomms9079 PMC4560802

[88] J.‐C. Tan , B. Civalleri , A. Erba , E. Albanese , CrystEngComm 2015, 17, 375.

[89] Y. Sakata , S. Furukawa , M. Kondo , K. Hirai , N. Horike , Y. Takashima , H. Uehara , N. Louvain , M. Meilikhov , T. Tsuruoka , S. Isoda , W. Kosaka , O. Sakata , S. Kitagawa , Science 2013, 339, 193. 23307740 10.1126/science.1231451

[90] D. A. Reed , B. K. Keitz , J. Oktawiec , J. A. Mason , T. Runčevski , D. J. Xiao , L. E. Darago , V. Crocellà , S. Bordiga , J. R. Long , Nature 2017, 550, 96. 28892810 10.1038/nature23674

[91] Y.‐P. Wang , X.‐G. Li , S.‐L. Liu , J. N. Fry , H.‐P. Cheng , Phys. Rev. B 2018, 97, 115419.

[92] C.‐W. Kung , T. C. Wang , J. E. Mondloch , D. Fairen‐Jimenez , D. M. Gardner , W. Bury , J. M. Klingsporn , J. C. Barnes , R. Van Duyne , J. F. Stoddart , M. R. Wasielewski , O. K. Farha , J. T. Hupp , Chem. Mater. 2013, 25, 5012.

[93] P. Jain , A. Stroppa , D. Nabok , A. Marino , A. Rubano , D. Paparo , M. Matsubara , H. Nakotte , M. Fiebig , S. Picozzi , E. S. Choi , A. K Cheetham , C. Draxl , N. S Dalal , V. S Zapf , npj Quantum Mater. 2016, 1, 16012.

[94] A. P. Katsoulidis , D. Antypov , G. F. S. Whitehead , E. J. Carrington , D. J. Adams , N. G. Berry , G. R. Darling , M. S. Dyer , M. J. Rosseinsky , Nature 2019, 565, 213. 30626943 10.1038/s41586-018-0820-9

[95] D.‐W. Fu , W. Zhang , R.‐G. Xiong , Dalton Trans. 2008, 30, 3946. 10.1039/b806255b 18648695

[96] S.‐I. Ohkoshi , K. Imoto , Y. Tsunobuchi , S. Takano , H. Tokoro , Nat. Chem. 2011, 3, 564. 21697879 10.1038/nchem.1067

[97] F. Tan , A. López‐Periago , M. E. Light , J. Cirera , E. Ruiz , A. Borrás , F. Teixidor , C. Viñas , C. Domingo , J. G. Planas , Adv. Mater. 2018, 30, 1800726. 10.1002/adma.201800726 29845666

[98] K. Müller , J. Helfferich , F. Zhao , R. Verma , A. B. Kanj , V. Meded , D. Bléger , W. Wenzel , L. Heinke , Adv. Mater. 2018, 30, 1706551. 10.1002/adma.201706551 29315923

[99] A. Modrow , D. Zargarani , R. Herges , N. Stock , Dalton Trans. 2011, 40, 4217. 21394353 10.1039/c0dt01629b

[100] R. Lyndon , K. Konstas , B. P. Ladewig , P. D. Southon , C. J. Kepert , M. R. Hill , Angew. Chem., Int. Ed. 2013, 52, 3695. 10.1002/anie.201206359 23401101

[101] J. Park , D. Yuan , K. T. Pham , J. R. Li , A. Yakovenko , H. C. Zhou , J. Am. Chem. Soc. 2012, 134, 99. 22148550 10.1021/ja209197f

[102] N. Yanai , T. Uemura , M. Inoue , R. Matsuda , T. Fukushima , M. Tsujimoto , S. Isoda , S. Kitagawa , J. Am. Chem. Soc. 2012, 134, 4501. 22372403 10.1021/ja2115713

[103] E. A. Dolgopolova , V. A. Galitskiy , C. R. Martin , H. N. Gregory , B. J. Yarbrough , A. M. Rice , A. A. Berseneva , O. A. Ejegbavwo , K. S. Stephenson , P. Kittikhunnatham , S. G. Karakalos , M. D. Smith , A. B. Greytak , S. Garashchuk , N. B. Shustova , J. Am. Chem. Soc. 2019, 141, 5350. 30840822 10.1021/jacs.8b13853

[104] S. Garg , H. Schwartz , M. Kozlowska , A. B. Kanj , K. Müller , W. Wenzel , U. Ruschewitz , L. Heinke , Angew. Chem., Int. Ed. 2019, 58, 1193. 10.1002/anie.201811458 30421842

[105] S. Steinwand , Z. Yu , S. Hecht , J. Wachtveitl , J. Am. Chem. Soc. 2016, 138, 12997. 27598007 10.1021/jacs.6b07720

[106] M. L. Aubrey , B. M. Wiers , S. C. Andrews , T. Sakurai , S. E. Reyes‐Lillo , S. M. Hamed , C.‐J. Yu , L. E. Darago , J. A. Mason , J.‐O. Baeg , F. Grandjean , G. J. Long , S. Seki , J. B. Neaton , P. Yang , J. R. Long , Nat. Mater. 2018, 17, 625. 29867169 10.1038/s41563-018-0098-1

[107] S. M. Yoon , S. C. Warren , B. A. Grzybowski , Angew. Chem. 2014, 126, 4526; 10.1002/anie.201309642 24633993

[108] G. Ding , Y. Wang , G. Zhang , K. Zhou , K. Zeng , Z. Li , Y. Zhou , C. Zhang , X. Chen , S.‐T. Han , Adv. Funct. Mater. 2019, 29, 1806637.

[109] L. Pan , Z. Ji , X. Yi , X. Zhu , X. Chen , J. Shang , G. Liu , R.‐W. Li , Adv. Funct. Mater. 2015, 25, 2677.

[110] J. M. Zadrozny , A. T. Gallagher , T. D. Harris , D. E. Freedman , J. Am. Chem. Soc. 2017, 139, 7089. 28453274 10.1021/jacs.7b03123

[111] A. Urtizberea , E. Natividad , P. J. Alonso , M. A. Andrés , I. Gascón , M. Goldmann , O. Roubeau , Adv. Funct. Mater. 2018, 28, 1801695.

[112] D. B. Cassidy , Eur. Phys. J. D 2018, 72, 53.

[113] D. Dutta , J. I. Feldblyum , D. W. Gidley , J. Imirzian , M. Liu , A. J. Matzger , R. S. Vallery , A. G. Wong‐Foy , Phys. Rev. Lett. 2013, 110, 197403. 23705740 10.1103/PhysRevLett.110.197403

[114] P. Crivelli , D. Cooke , B. Barbiellini , B. L. Brown , J. I. Feldblyum , P. Guo , D. W. Gidley , L. Gerchow , A. J. Matzger , Phys. Rev. B 2014, 89, 241103.

[115] A. C. L. Jones , H. J. Goldman , Q. Zhai , P. Feng , H. W. K. Tom , A. P. Mills Jr. , Phys. Rev. Lett. 2015, 114, 153201. 25933312 10.1103/PhysRevLett.114.153201

[116] F. P. Doty , C. A. Bauer , A. J. Skulan , P. G. Grant , M. D. Allendorf , Adv. Mater. 2009, 21, 95.

[117] S. R. Mathis II , S. T. Golafale , J. Bacsa , A. Steiner , C. W. Ingram , F. P. Doty , E. Auden , K. Hattar , Dalton Trans. 2017, 46, 491. 27966707 10.1039/c6dt03755k

[118] S. R. Mathis II , S. T. Golafale , K. M. Solntsev , C. W. Ingram , Crystals 2018, 8, 53.

[119] J. Lu , X.‐H. Xin , Y.‐J. Lin , S.‐H. Wang , J.‐G. Xu , F.‐K. Zheng , G.‐C. Guo , Dalton Trans. 2019, 48, 1722. 30637431 10.1039/c8dt04587a

[120] I. Mihalcea , N. Henry , T. Bousquet , C. Volkringer , T. Loiseau , Cryst. Growth Des. 2012, 12, 4641.

[121] R. C. Severance , S. A. Vaughn , M. D. Smith , H.‐C. zur Loye , Solid State Sci. 2011, 13, 1344.

[122] L. A. Borkowski , C. L. Cahill , Cryst. Growth Des. 2006, 6, 2241.

[123] Y. Li , Z. Weng , Y. Wang , L. Chen , D. Sheng , J. Diwu , Z. Chai , T. E. Albrecht‐Schmitt , S. Wang , Dalton Trans. 2016, 45, 918. 26672441 10.1039/c5dt04183j

[124] J. Andreo , E. Priola , G. Alberto , P. Benzi , D. Marabello , D. M. Proserpio , C. Lamberti , E. Diana , J. Am. Chem. Soc. 2018, 140, 14144. 30285430 10.1021/jacs.8b07113

[125] Y. Feng , H. Wang , S. Zhang , Y. Zhao , J. Gao , Y. Zheng , P. Zhao , Z. Zhang , M. J. Zaworotko , P. Cheng , S. Ma , Y. Chen , Adv. Mater. 2019, 31, 1805148. 10.1002/adma.201805148 30480344

[126] K. E. deKrafft , W. S. Boyle , L. M. Burk , O. Z. Zhou , W. Lin , J. Mater. Chem. 2012, 22, 18139. 23049169 10.1039/C2JM32299D PMC3462458

[127] D. L. Bailey , D. W. Townsend , P. E. Valk , M. N. Maisey , Positron Emission Tomography, Springer‐Verlag London Ltd, London 2005.

[128] D. Chen , D. Yang , C. A. Dougherty , W. Lu , H. Wu , X. He , T. Cai , M. E. Van Dort , B. D. Ross , H. Hong , ACS Nano 2017, 11, 4315. 28345871 10.1021/acsnano.7b01530 PMC5477053

[129] R. Lakes , Nature 1993, 361, 511.

[130] J. Bauer , L. R. Meza , T. A. Schaedler , R. Schwaiger , X. Zheng , L. Valdevit , Adv. Mater. 2017, 29, 1701850. 10.1002/adma.201701850 28873250

[131] X. Yu , J. Zhou , H. Liang , Z. Jiang , L. Wu , Prog. Mater. Sci. 2018, 94, 114.

[132] J.‐H. Lee , J. P. Singer , E. L. Thomas , Adv. Mater. 2012, 24, 4782. 22899377 10.1002/adma.201201644

[133] A. Carné‐Sánchez , I. Imaz , K. C. Stylianou , D. Maspoch , Chem. ‐ Eur. J. 2014, 20, 5192. 24643892 10.1002/chem.201304529

[134] J. Reboul , S. Furukawa , N. Horike , M. Tsotsalas , K. Hirai , H. Uehara , M. Kondo , N. Louvain , O. Sakata , S. Kitagawa , Nat. Mater. 2012, 11, 717. 22728321 10.1038/nmat3359

[135] C. Avci , I. Imaz , A. Carné‐Sánchez , J. A. Pariente , N. Tasios , J. Pérez‐Carvajal , M. I. Alonso , A. Blanco , M. Dijkstra , C. López , D. Maspoch , Nat. Chem. 2018, 10, 78. 10.1038/nchem.2875 29256498

[136] J. Cui , N. Gao , C. Wang , W. Zhu , J. Li , H. Wang , P. Seidel , B. J. Ravoo , G. Li , Nanoscale 2014, 6, 11995. 25177919 10.1039/c4nr03095h

[137] O. Dalstein , E. Gkaniatsou , C. Sicard , O. Sel , H. Perrot , C. Serre , C. Boissière , M. Faustini , Angew. Chem. 2017, 129, 14199; 10.1002/anie.201706745 28940925

[138] K. Shen , L. Zhang , X. Chen , L. Liu , D. Zhang , Y. Han , J. Chen , J. Long , R. Luque , Y. Li , B. Chen , Science 2018, 359, 206. 29326271 10.1126/science.aao3403

[139] Y.‐N. Wu , F. Li , W. Zhu , J. Cui , C.‐A. Tao , C. Lin , P. M. Hannam , G. Li , Angew. Chem. 2011, 123, 12726; 10.1002/anie.201104597 22006864

[140] N. Yanai , S. Granick , Angew. Chem. 2012, 124, 5736; 10.1002/anie.201109132 22544724

[141] M. Pang , A. J. Cairns , Y. Liu , Y. Belmabkhout , H. C. Zeng , M. Eddaoudi , J. Am. Chem. Soc. 2012, 134, 13176. 22812681 10.1021/ja3049282

[142] P. Falcaro , K. Okada , T. Hara , K. Ikigaki , Y. Tokudome , A. W. Thornton , A. J. Hill , T. Williams , C. Doonan , M. Takahashi , Nat. Mater. 2017, 16, 342. 27918565 10.1038/nmat4815

[143] B. Chang , Y. Yang , H. Jansen , F. Ding , K. Mølhave , H. Sun , Adv. Mater. Interfaces 2018, 5, 1701270.

[144] A. I. Kuznetsov , A. E. Miroshnichenko , M. L. Brongersma , Y. S. Kivshar , B. Luk'yanchuk , Science 2016, 354, aag2472. 27856851 10.1126/science.aag2472

[145] L. Mingabudinova , A. S. Zalogina , A. Krasilin , M. I. Petrova , P. Trofimov , Y. A. Mezenov , E. Ubyivovk , P. Lönnecke , A. Nomine , J. Ghanbaja , T. Belmonte , V. A. Milichko , Nanoscale 2019, 11, 10155. 31038502 10.1039/c9nr02167a

[146] K. E. Evans , A. Alderson , Adv. Mater. 2000, 12, 617.

[147] C. Huang , L. Chen , Adv. Mater. 2016, 28, 8079. 27378610 10.1002/adma.201601363

[148] G. N. Greaves , A. L. Greer , R. S. Lakes , T. Rouxel , Nat. Mater. 2011, 10, 823. 22020006 10.1038/nmat3134

[149] M. R. Ryder , J.‐C. Tan , Dalton Trans. 2016, 45, 4154. 26426139 10.1039/c5dt03514g

[150] S. Kamrava , D. Mousanezhad , H. Ebrahimi , R. Ghosh , A. Vaziri , Sci. Rep. 2017, 7, 46046. 28387345 10.1038/srep46046 PMC5384242

[151] K. Bertoldi , V. Vitelli , J. Christensen , M. van Hecke , Nat. Rev. Mater. 2017, 2, 17066.

[152] M. R. Ryder , B. Civalleri , J.‐C. Tan , Phys. Chem. Chem. Phys. 2016, 18, 9079. 26972778 10.1039/c6cp00864j

[153] M. R. Ryder , B. Civalleri , G. Cinque , J.‐C. Tan , CrystEngComm 2016, 18, 4303.

[154] R. Gatt , L. Mizzi , J. I. Azzopardi , K. M. Azzopardi , D. Attard , A. Casha , J. Briffa , J. N. Grima , Sci. Rep. 2015, 5, 8395. 25670400 10.1038/srep08395 PMC4323639

[155] J. I. Lipton , R. MacCurdy , Z. Manchester , L. Chin , D. Cellucci , D. Rus , Science 2018, 360, 632. 29748279 10.1126/science.aar4586

[156] A. D. Fortes , E. Suard , K. S. Knight , Science 2011, 331, 742. 21311017 10.1126/science.1198640

[157] I. E. Collings , M. G. Tucker , D. A. Keen , A. L. Goodwin , CrystEngComm 2014, 16, 3498.

[158] M. Kadic , T. Bückmann , N. Stenger , M. Thiel , M. Wegener , Appl. Phys. Lett. 2012, 100, 191901.

[159] A. U. Ortiz , A. Boutin , A. H. Fuchs , F.‐X. Coudert , Phys. Rev. Lett. 2012, 109, 195502. 23215398 10.1103/PhysRevLett.109.195502

[160] Y. Yan , A. E. O'Connor , G. Kanthasamy , G. Atkinson , D. R. Allan , A. J. Blake , M. Schröder , J. Am. Chem. Soc. 2018, 140, 3952. 29394049 10.1021/jacs.7b11747

[161] J. Binns , K. V. Kamenev , K. E. R. Marriott , G. J. McIntyre , S. A. Moggach , M. Murrie , S. Parsons , Chem. Commun. 2016, 52, 7486. 10.1039/c6cc02489k 27203683

[162] P. Serra‐Crespo , A. Dikhtiarenko , E. Stavitski , J. Juan‐Alcañiz , F. Kapteijn , F.‐X. Coudert , J. Gascon , CrystEngComm 2015, 17, 276. 25722647 10.1039/C4CE00436A PMC4338503

[163] T. A. Schaedler , A. J. Jacobsen , A. Torrents , A. E. Sorensen , J. Lian , J. R. Greer , L. Valdevit , W. B. Carter , Science 2011, 334, 962. 22096194 10.1126/science.1211649

[164] R. Sanz , F. Martínez , G. Orcajo , L. Wojtas , D. Briones , Dalton Trans. 2013, 42, 2392. 23208471 10.1039/c2dt32138f

[165] E. J. García , J. P. S. Mowat , P. A. Wright , J. Pérez‐Pellitero , C. Jallut , G. D. Pirngruber , J. Phys. Chem. C 2012, 116, 26636.

[166] Z. C. Eckel , C. Zhou , J. H. Martin , A. J. Jacobsen , W. B. Carter , T. A. Schaedler , Science 2016, 351, 58. 26721993 10.1126/science.aad2688

[167] T. Frenzel , M. Kadic , M. Wegener , Science 2017, 358, 1072. 29170236 10.1126/science.aao4640

[168] C. Coulais , Science 2017, 358, 994. 29170220 10.1126/science.aaq0818

[169] J. T. B. Overvelde , T. A. de Jong , Y. Shevchenko , S. A. Becerra , G. M. Whitesides , J. C. Weaver , C. Hoberman , K. Bertoldi , Nat. Commun. 2016, 7, 10929. 26965475 10.1038/ncomms10929 PMC4793042

[170] C. Coulais , A. Sabbadini , F. Vink , M. van Hecke , Nature 2018, 561, 512. 30258138 10.1038/s41586-018-0541-0

[171] F. X. Coudert , J. D. Evans , Coord. Chem. Rev. 2019, 388, 48.

[172] M. B. Pinson , M. Stern , A. C. Ferrero , T. A. Witten , E. Chen , A. Murugan , Nat. Commun. 2017, 8, 15477. 28516913 10.1038/ncomms15477 PMC5454341

[173] W. Cai , A. Gładysiak , M. Anioła , V. J. Smith , L. J. Barbour , A. Katrusiak , J. Am. Chem. Soc. 2015, 137, 9296. 25945394 10.1021/jacs.5b03280

[174] W. Cai , A. Katrusiak , Nat. Commun. 2014, 5, 4337. 24993679 10.1038/ncomms5337

[175] Z. G. Nicolaou , A. E. Motter , Nat. Mater. 2012, 11, 608. 22609557 10.1038/nmat3331

[176] J. Heinen , A. D. Ready , T. D. Bennett , D. Dubbeldam , R. W. Friddle , N. C. Burtch , ACS Appl. Mater. Interfaces 2018, 10, 21079. 29873475 10.1021/acsami.8b06604

[177] Y.‐R. Miao , Z. Su , K. S. Suslick , J. Am. Chem. Soc. 2017, 139, 4667. 28328219 10.1021/jacs.7b01593

[178] C. L. Hobday , R. J. Marshall , C. F. Murphie , J. Sotelo , T. Richards , D. R. Allan , T. Düren , F. X. Coudert , R. S. Forgan , C. A. Morrison , S. A. Moggach , T. D. Bennett , Angew. Chem., Int. Ed. 2016, 55, 2401. 10.1002/anie.201509352 PMC5021150 26797762

[179] T. D. Bennett , A. K. Cheetham , A. H. Fuchs , F. X. Coudert , Nat. Chem. 2017, 9, 11. 10.1038/nchem.2691 27995920

[180] X. Zhou , Y.‐R. Miao , W. L. Shaw , K. S. Suslick , D. D. Dlott , J. Am. Chem. Soc. 2019, 141, 2220. 30700090 10.1021/jacs.8b12905

